# Altered Insulin Signaling in Alzheimer’s Disease Brain – Special Emphasis on PI3K-Akt Pathway

**DOI:** 10.3389/fnins.2019.00629

**Published:** 2019-06-18

**Authors:** Sami Gabbouj, Simo Ryhänen, Mikael Marttinen, Rebekka Wittrahm, Mari Takalo, Susanna Kemppainen, Henna Martiskainen, Heikki Tanila, Annakaisa Haapasalo, Mikko Hiltunen, Teemu Natunen

**Affiliations:** ^1^Institute of Biomedicine, University of Eastern Finland, Kuopio, Finland; ^2^A.I. Virtanen Institute for Molecular Sciences, University of Eastern Finland, Kuopio, Finland

**Keywords:** Alzheimer’s disease, type 2 diabetes, insulin, phosphatidylinositol-4, 5-bisphosphate 3-kinase (PI3K), Akt (Protein kinase B, PKB), glucose metabolism, neuroinflammation, autophagy

## Abstract

Alzheimer’s disease (AD) and type 2 diabetes (T2D) are both diseases with increasing prevalence in aging populations. T2D, characterized by insulin resistance and defective insulin signaling, is a common co-morbidity and a risk factor for AD, increasing the risk approximately two to fourfold. Insulin exerts a wide variety of effects as a growth factor as well as by regulating glucose, fatty acid, and protein metabolism. Certain lifestyle factors, physical inactivity and typical Western diet (TWD) containing high fat and high sugar are strongly associated with insulin resistance and T2D. The PI3K-Akt signaling pathway is a major mediator of effects of insulin and plays a crucial role in T2D pathogenesis. Decreased levels of phosphatidylinositol-4,5-bisphosphate 3-kinase (PI3K) subunits as well as blunted Akt kinase phosphorylation have been observed in the AD brain, characterized by amyloid-β and tau pathologies. Furthermore, AD mouse models fed with TWD have shown to display altered levels of PI3K subunits. How impaired insulin-PI3K-Akt signaling in peripheral tissues or in the central nervous system (CNS) affects the development or progression of AD is currently poorly understood. Interestingly, enhancement of PI3K-Akt signaling in the CNS by intranasal insulin (IN) treatment has been shown to improve memory *in vivo* in mice and in human trials. Insulin is known to augment neuronal growth and synapse formation through the PI3K-Akt signaling pathway. However, PI3K-Akt pathway mediates signaling related to different functions also in other cell types, like microglia and astrocytes. In this review, we will discuss the most prominent molecular mechanisms related to the PI3K-Akt pathway in AD and how T2D and altered insulin signaling may affect the pathogenesis of AD.

## Introduction

Both Alzheimer’s disease (AD) and type 2 diabetes (T2D) are diseases reaching epidemic proportions. The main neuropathological findings in AD, the most common form of dementia, are β-amyloid plaques, composed of extracellular aggregates of the β-amyloid (Aβ) peptide, and intracellular neurofibrillary tangles (NFT) formed of hyperphosphorylated tau protein (Hardy and Selkoe, 2002). However, there are several other pathological features related to AD, including loss of synapses and neurons, inflammatory activation of microglia and astrocytes as well as impairment in glucose metabolism and insulin-phosphatidylinositol-4,5-bisphosphate 3-kinase (PIK3)-Akt signaling in the brain (Mosconi et al., 2008; Serrano-Pozo et al., 2011; Talbot et al., 2012).

Type 2 diabetes is a complex, age- and lifestyle-related chronic disease. It is characterized by increased glucose and insulin levels in the blood, insulin resistance, metabolic abnormalities, and chronic inflammation (Sjöholm and Nyström, 2006; Ashcroft and Rorsman, 2012). T2D is one of the most important co-morbidities of AD, increasing the risk of AD two to fourfold (Craft, 2007; Sims-Robinson et al., 2010). Lifestyle factors, such as physical inactivity and excess calories gained from the typical Western diet (TWD), play a central role in T2D (Khazrai et al., 2014). Several *in vivo* studies in AD mouse models have shown that TWD induces T2D phenotype and exacerbates memory impairments, which are linked to altered PI3K-Akt signaling in the brain (Kang et al., 2017; Kothari et al., 2017; Salas et al., 2018). Insulin is a crucial factor controlling blood glucose levels and it facilitates cellular glucose uptake in peripheral tissues by activating the PI3K-Akt pathway (Kim and Feldman, 2012). In the brain, insulin does not have a major role in glucose metabolism. However, insulin and the PI3K-Akt signaling pathway play a significant role in neuronal health as well as synapse formation and maintenance (Van Der Heide et al., 2005; Chiu et al., 2008; Lee et al., 2011). Apart from neurons, PI3K-Akt signaling pathway also plays a central role in other cell types in the brain and TWD has been shown to affect, e.g., the function of microglia (Spagnuolo et al., 2015; Spencer et al., 2019).

Imaging studies have shown that T2D is often associated with changes in the brain that are typically detected in patients with AD and related dementias, including decreased hippocampal volume, reduced glucose metabolism, and changes in cerebral blood flow (Baker et al., 2011; Moran et al., 2013; Willette et al., 2015). Furthermore, alterations in the insulin signaling pathway as well as decreased levels of insulin and insulin receptors (IR) have been observed in the AD brain (Steen et al., 2005; Talbot et al., 2012). Similarly, to T2D, other abnormalities such as metabolic stress and inflammation are also characteristic in AD (Moloney et al., 2010; Talbot et al., 2012). However, the relationship between T2D and the main neuropathological finding in AD, cerebral Aβ accumulation, remains unclear. While most of the studies have reported no association, two studies have found a significant correlation between peripheral insulin resistance and brain Aβ levels as measured by Pittsburgh compound B-positron emission tomography (PiB-PET) (Willette et al., 2015; Ekblad et al., 2018). The impaired insulin-PI3K-Akt signaling observed in the AD brain has led to clinical trials studying whether the enhancement of this pathway using intranasal insulin (IN) treatment is beneficial. Intranasally administered insulin reaches the brain via the olfactory and trigeminal nerves (Lochhead et al., 2019). Further, doses up to 40 IU in elderly subjects seem not to induce systemic hypoglycemia (Craft et al., 2012). The majority of results from studies in cognitively healthy humans as well as in AD patients are encouraging, suggesting that IN enhances memory and cognition (reviewed by Chapman et al., 2017). However, the effects appear to depend on the dose and the dose regime (acute vs. repeated), cognitive test used, and the *APOE* genotype. Interestingly, in a preclinical APP/PS1 AD mouse model, IN treatment led to the specific activation of the Akt2 isoform (Gabbouj et al., 2019). This suggests that Akt kinases may have isoform-specific roles in insulin signaling in the brain. Furthermore, the same study revealed differential effects of IN on the expression profile of homeostatic microglia and the markers of autophagy in the hippocampus of WT and APP/PS1 mice.

Despite the strong epidemiological association between AD and T2D, the underlying molecular mechanisms are still not fully understood. It is probable that T2D affects the development and progression of AD and related disorders via several mechanisms, some of which may be directly or indirectly linked to the insulin-PI3K-Akt signaling pathway. The aim in this review article is to summarize and discuss the effects of altered insulin-PI3K-Akt signaling and T2D on the pathogenesis of AD.

## Glucose Metabolism and Insulin Resistance in the Brain

Common findings in T2D are hyperglycemia and insulin resistance, meaning that peripheral tissues do not respond normally to insulin in order to take up glucose from the blood. Insulin resistance can be clinically assessed using, e.g., the homeostatic model assessment for insulin resistance (HOMA-IR) from fasting insulin and glucose levels (Matthews et al., 1985). However, the definition of insulin resistance in the brain is not as clear. While glucose uptake in peripheral tissues is heavily dependent on insulin, glucose uptake in the brain is mainly independent of insulin (Kim and Feldman, 2012). The term “brain insulin resistance” has been used to provide an underlying reason for the glucose hypometabolism observed in the AD brain. However, since insulin does not play a major role in brain glucose metabolism, insulin resistance in the brain is considered as an impairment in the insulin signaling pathway.

**FIGURE 1:**
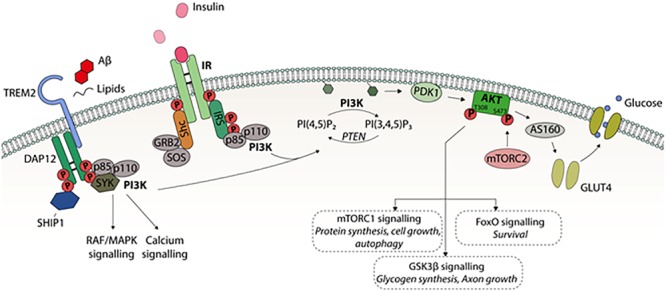
A schematic presentation of PI3K-Akt intracellular signaling. Different extracellular stimuli, e.g., growth factors mediate their effects by receptors belonging to receptor tyrosine kinase (RTK) family locating in the cell membrane. Binding of insulin to IR leads to activating tyrosine phosphorylation of insulin receptor substrate (IRS). This activates phosphatidylinositol-4,5-bisphosphate 3-kinase (PI3K) consisting of regulatory p85 and catalytic p110 subunits. PI3K converts phosphatidylinositol (3,4)-bisphosphate (PIP_2_) into phosphatidylinositol (3,4,5)-trisphosphate (PIP_3_), which recruits Akt-kinase to cell membrane. PIP_3_ activates phosphoinositide-dependent protein kinase 1 (PDK1) to phosphorylate threonine 308 site in Akt1. For the full activation of Akt, serine 473 phosphorylation by mammalian target for rapamycin complex 2 (mTORC2) is needed. PI3K-Akt pathway regulates several cellular functions via downstream factors; Akt substrate 160 kDa (AS160) controls insulin dependently on glucose transporter 4 (GLUT4) translocation to the cell membrane and glucose uptake into the cell, mTORC1 regulates autophagy, protein synthesis, and cell growth, glycogen synthase kinase 3 (GSK3) affects glycogen synthesis, axon growth, and tau phosphorylation, and forkhead box (FOX) transcription factors regulate many functions, such as cell survival. In microglia, cell surface receptor TREM2 signaling regulates the phagocytosis, motility, autophagy, survival, and proliferation. TREM2 is activated by several ligands including phospholipids, lipoproteins, and oligomeric Aβ leading to interaction with activating adaptor protein DAP12. This in turn, leads to activation of PI3K-Akt pathway.

Glucose uptake in peripheral tissues is based on the insulin-dependent glucose transporter 4 (GLUT4) (Huang and Czech, 2007). Insulin activates the PI3K-Akt pathway and the activated Akt kinase subsequently phosphorylates Akt substrate 160 kDa (AS160), which recruits GLUT4 to the plasma membrane, allowing glucose to efficiently enter the cell (Figure 1). In the brain, endothelial cells and astrocytes, which are part of the blood-brain barrier (BBB), express mainly GLUT1, while the most common glucose transporter in neurons is GLUT3. Both GLUT1 and GLUT3 are insulin-independent. However, it has been shown that insulin-dependent GLUT4 is expressed to some extent in several brain regions, such as hippocampus, cerebellum, and olfactory bulb (Vannucci et al., 1998).

Recently, a rare P50T variant in *AKT2* gene was shown to increase the risk of T2D in humans as well as the fasting levels of insulin on average ∼15% as compared to matched controls (Manning et al., 2017). Interestingly, the carriers of this variant showed decreased glucose uptake in the peripheral tissues, such as skeletal muscle, whereas glucose uptake in the brain was elevated ∼20% upon hyperinsulinemic–euglycemic clamp (Latva-Rasku et al., 2018). Given these observations, further studies are needed to uncover whether this genetic variation affects cognitive performance at the higher age or the risk of dementia.

## Insulin-PI3K-Akt Signaling in the Brain

Insulin, a hormone produced in the pancreas, has a wide variety of functions. Of these, the best known is the regulation of glucose uptake into peripheral tissues, such as skeletal muscle, liver, and adipose tissue (Dimitriadis et al., 2011). Insulin is able to penetrate the BBB via a saturable transport system (Banks et al., 2012), meaning that increased serum insulin levels elevate those of brain insulin only to a certain level.

Insulin transport varies between different brain regions. The olfactory bulb is reported to have the highest rate of insulin transport, probably due to having the highest concentration of IRs (Hill et al., 1986; Gupta et al., 1992). In general, IRs are more abundantly expressed in neurons as compared to other cell types in the brain (Frölich et al., 1998). IRs have been observed in all compartments of neurons, particularly in dendrites and presynaptic terminals, emphasizing their role in dendritic growth and synapse formation (Abbott et al., 1999; Lee et al., 2011).

The binding of insulin to the IR can activate two distinct branches of insulin signaling: the Ras-mitogen-activated protein kinase (MAPK) and PI3K-Akt pathways (Figure 1; Kim and Feldman, 2012). The IR and the insulin-like growth factor 1 (IGF1) receptor (IGF1R) are the major activators of PI3K. IR and IGF1R belong to the receptor tyrosine kinase (RTK) family, which includes receptors for many growth factors, such as vascular endothelial growth factor (VEGF) and nerve growth factor (NGF) (Lemmon and Schlessinger, 2010). Furthermore, the different RTKs are able to activate the same intracellular PI3K-Akt pathway as insulin. The binding of insulin causes a conformational change in the IR, inducing autophosphorylation followed by the recruitment of intracellular insulin receptor substrate (IRS) proteins, which are subsequently tyrosine-phosphorylated. While the phosphorylation of specific tyrosine residues activates IRS, there are serine phosphorylation sites that inactivate IRSs by causing their dissociation from the IR and decreasing tyrosine phosphorylation (Copps and White, 2012). Tyrosine-phosphorylated IRS activates PI3Ks, a kinase family involved in several intracellular signaling transduction processes.

PI3K is a heteromeric protein consisting of a p110 catalytic subunit and a p85 regulatory subunit (Figure 1). The SH2 domain of the PI3K p85 subunit binds to the phosphotyrosines in the cytosolic domain of the plasma membrane-resident RTKs. P110 converts phosphatidylinositol (3,4)-bisphosphate (PIP_2_) into phosphatidylinositol (3,4,5)-trisphosphate (PIP_3_), leading to the activation of numerous downstream kinases, such as Akt (Boura-Halfon and Zick, 2009).

Akt is a family of serine/threonine kinases consisting of three isoforms: Akt1, Akt2, and Akt3. These isoforms exhibit different expression patterns depending on the brain region and cell type. For example, Akt1 and Akt3 are distributed throughout the somatic layers of the hippocampus, while Akt2 is mostly expressed in astrocytes but not in neurons of the hippocampus (Levenga et al., 2017). Once activated, Akt regulates cell survival, proliferation, cytoskeletal organization, cell metabolism, vesicle trafficking, and glucose transport (Noguchi and Suizu, 2012). Akt is activated upon its interaction with the pleckstrin homology (PH)-domain of PIP_3_ allowing phosphoinositide-dependent protein kinase 1 (PDK1) to phosphorylate threonine 308/309/305 of Akt1/2/3, respectively, at the plasma membrane. Full activation of Akt also requires the phosphorylation of serine 473/474/472 of Akt1/2/3, respectively. The kinase responsible for the serine phosphorylation of Akt is mammalian target for rapamycin complex 2 (mTORC2), although the exact mechanism of this mTORC2-mediated activation is still unclear (Noguchi and Suizu, 2012). PI3K/Akt regulates downstream factors, such as glycogen synthase kinase 3 (GSK3), mTORC1, and forkhead box (FOX) transcription factors, affecting a plethora of cellular functions in peripheral tissues and in the brain (Figure 1; Kim and Feldman, 2012).

## Alterations of PI3K-Akt Signaling in the AD Brain

In the AD brain, alterations in the PI3K-Akt pathway primarily manifest as decreased phosphorylation or total levels of the components in the insulin-PI3K-Akt signaling cascade (Steen et al., 2005; Liu et al., 2011). Previous studies have found that Aβ oligomers inhibit the PI3K-Akt pathway, which leads to neuronal death. Post-mortem analysis of different AD brain regions has revealed reduced levels of insulin, IR, IGF1, and IGF1R (Steen et al., 2005; Liu et al., 2011). In addition, the analysis of post-mortem AD brain samples showed decreased levels of PI3K subunits (both p85 and p110) and reduced phosphorylation of Akt and GSK3β (Steen et al., 2005; Moloney et al., 2010). Interestingly, these changes were associated with several important pathological hallmarks of AD, such as the NFT pathology as well as microglial and astroglial markers (Rivera et al., 2005). Progression of NFT pathology in AD brain from one brain region to another during the disease course exhibits a certain chronological pattern, which is defined by Braak staging and correlates relatively well with clinical dementia symptoms (Braak et al., 2006). GSK3β is one of the most important tau-phosphorylating kinases (Wilson et al., 2013). PI3K-Akt signaling regulates GSK3β by phosphorylating the serine 9 residue, which inhibits GSK3β activity. In cultured neurons, insulin and IGF1 have been shown to decrease tau phosphorylation through Akt-mediated GSK3β inhibition (Hong and Lee, 1997). Talbot et al. (2012) subjected hippocampal tissue from normal post-mortem brains and from AD brains to *ex vivo* insulin stimulation with physiological doses. The normal tissue responded strongly to insulin as measured by the enhanced phosphorylation of IRS-1, Akt, GSK3α, and GSK3β. In contrast, the AD hippocampal tissue demonstrated drastically reduced insulin-mediated downstream activation (Talbot et al., 2012). Interestingly, two separate studies showed abnormal basal phosphorylation levels of proteins in the insulin-IRS-1-Akt pathway in post-mortem AD brains. Furthermore, these changes correlated positively with Aβ and tau lesions and negatively with memory and global cognition scores. Intriguingly, hippocampal insulin resistance contributed to the presence of Aβ and tau lesions independently of cognitive impairment (Bomfim et al., 2012; Talbot et al., 2012).

Disturbances in autophagy play a significant role in many neurodegenerative diseases, including AD, which is characterized by the accumulation of toxic intracellular protein aggregates (Son et al., 2012). mTOR, a key regulator of autophagy induction, is a central protein in two complexes, mTORC1 and mTORC2, which are both important downstream factors in the PI3K-Akt signaling pathway. As mentioned above, mTORC2 activates Akt by phosphorylating it at serine 473, while Akt activates mTORC1. In turn, active mTORC1 inhibits the induction of autophagy and promotes protein synthesis in neurons (Stoica et al., 2011), making mTORC1 a crucial factor regulating the balance between autophagy and protein synthesis. Different stimuli, such as insulin, IGFs, growth factors, and amino acids activate the PI3K-Akt-mTORC1 pathway and inhibit autophagy, while e.g., starvation inactivates this pathway, leading to increased autophagy. Constitutive autophagy is considered highly efficient in healthy neurons. Neurons in the AD brain display large amounts of autophagosomal vesicles (Boland et al., 2008) but it is not currently clear whether this results from decreased activation mTORC1, leading to increased autophagosome formation, or from defects in the later steps of autophagy, such as impairment in the clearance of autophagosomes through lysosomes (Boland et al., 2008).

### Neuroinflammation and PI3K-Akt Signaling in AD Brain

Chronic, low-grade inflammation is one of the main features observed in both T2D (Calle and Fernandez, 2012) and AD as well as other neurodegenerative diseases (Heneka et al., 2015). Elevated levels of inflammatory markers, such as tumor necrosis factor alpha (TNFα) and other cytokines, have been observed in brain and in blood samples of AD patients (Perry et al., 2010; Swardfager et al., 2010) and in peripheral tissues of subjects with T2D (Sjöholm and Nyström, 2006). TNFα plays a crucial role in peripheral insulin resistance. It activates c-Jun kinase (JNK), which leads to inhibitory serine phosphorylation of IRS and blockade of insulin signaling (Gregor and Hotamisligil, 2011). Aβ oligomers have also been shown to activate JNK, leading to inhibitory phosphorylation of IRS in hippocampal neurons of Aβ plaque producing transgenic mice (Bomfim et al., 2012). Importantly, this finding was also confirmed in post-mortem AD brains (Bomfim et al., 2012).

Microglia, the resident immune cells in the CNS, are responsible for neuroinflammation. High insulin levels promote inflammatory responses in the brain, based on increased TNFα, interleukin 1β and 6 (IL1β and IL6) levels observed in the CSF of healthy individuals after an acute dose of insulin (Craft, 2005; Fishel et al., 2005). In addition to the secretion of these pro-inflammatory cytokines, the same study showed that hyperinsulinemia increased Aβ levels in the plasma, suggesting that hyperinsulinemia can exacerbate neuroinflammation and provoke AD pathogenesis (Fishel et al., 2005). This may be explained by the competition of insulin and Aβ for degradation by the same enzyme, insulin degrading enzyme (Zhao, 2004).

There are two extremes in the spectrum of the classical activation status of microglia: M1 and M2. M2 represents the anti-inflammatory phenotype, characterized by the secretion of anti-inflammatory cytokines, such as IL10, while M1 is pro-inflammatory (Tang and Le, 2016). Microglia adopt the M1 phenotype when Toll-like receptor 4 (TLR4) is activated by ligands, e.g., lipopolysaccharide. This leads to activation and secretion of high levels of pro-inflammatory cytokines, such as TNFα, IL1β, IL6, and nitric oxide (NO). Activation of TLR4 triggers the PI3K-Akt-mTORC1 pathway, which in turn regulates nuclear factor-kappa B (NFκB), which controls transcription, cytokine production and cell survival in immune cells (Fang et al., 2017).

Secretion of inflammatory mediators TNFα, IL1α and complement component 1q (C1q), by activated microglia leads to inflammatory responses also in astrocytes and to transition from A2 to the neurotoxic A1 phenotype in astrocytes (Liddelow et al., 2017). Reactive A1 astrocytes lose their ability to support neuronal outgrowth and synaptogenesis leading to death of neurons. Interestingly, transition to A1 phenotype can be rescued by upregulating PI3K-Akt pathway (Xu et al., 2018).

Importantly, a recent study utilizing a single cell RNA sequencing technology revealed a novel disease-associated microglia (DAM) phenotype in amyloid plaque producing transgenic mice (Keren-Shaul et al., 2017). DAMs are a subset of microglia occurring also in other neurodegenerative diseases, such as amyotrophic lateral sclerosis (ALS), and they co-exist with Aβ plaques in AD (Keren-Shaul et al., 2017). DAM development is a two-step process where Trem2-PI3K-Akt pathway plays a central role. Stage 1 DAM transition is Trem2-independent, and factors driving this step are currently unknown.

At stage 1, there is a significant downregulation of homeostatic microglia genes, including *Cx3cr1, P2ry12*, and simultaneously increased expression of Trem2 regulators/adaptors Dap12 (Tyrobp) and Apoe. Interestingly, the levels of *Pik3r1* (p85) were decreased in microglia upon the transition from homeostatic to DAM phenotype (Keren-Shaul et al., 2017). Proceeding to stage 2 is Trem2-dependent and it is characterized by the elevated expression of certain genes such as *Trem2, Lpl, Cst7*, and *Clec7a*, which are involved in lysosomal, phagocytic and lipid metabolism pathways (Keren-Shaul et al., 2017; Deczkowska et al., 2018). In addition to enhanced phagocytic activity, production of proinflammatory cytokines is suppressed in microglia with the DAM phenotype (Ma et al., 2015).

TREM2 activation leads to DAP12 phosphorylation via Src family kinases, initiating the downstream signaling cascades including PI3K, PKC, and ERK. The PI3K-Akt signaling pathway has been shown to be downstream of Trem2-mediated signaling in microglia (Figure 1), since siRNA-mediated knockdown of Trem2 in microglia leads to decreased serine 473 phosphorylation of Akt and consequently decreased phosphorylation of GSK3β at serine 9 (Zheng et al., 2017). Additionally, it was recently shown that defective Trem2 signaling in microglia of Aβ plaque producing transgenic mice resulted in impaired Akt-mTORC1 signaling with simultaneous activation of AMP activated protein kinase. This led to the accumulation of autophagosomes, metabolic impairment, and further, decreased ability of microglia to form clusters around Aβ-plaques and increased formation of dystrophic neurites (Ulland et al., 2017). Thus, TREM2-DAP12 signaling utilizes at least partially the same intracellular PI3K-Akt signaling as insulin to induce its downstream effects.

## Concluding Remarks

**FIGURE 2:**
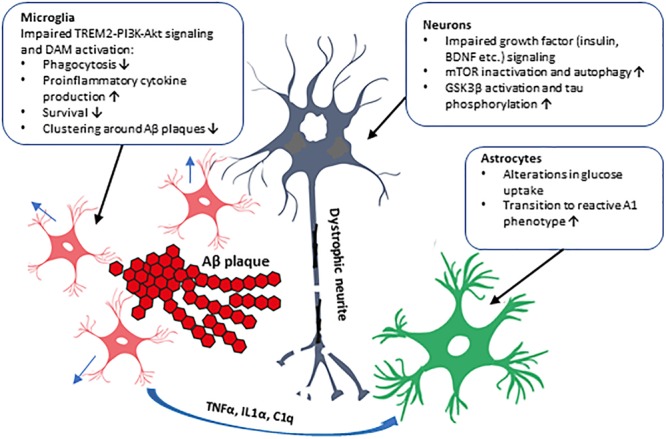
Schematic illustration of the cell type-specific effects of impaired PI3K-Akt signaling in AD brain. The impaired PI3K-Akt signaling might be due to decreased PI3K subunit levels observed in the brain of AD patients or due to T2D, which alters insulin levels and affects PI3K-Akt pathway in the brain, augmenting AD pathogenesis. These alterations could have distinct effects in different cell types in the brain, as shown in the figure. Activation of PI3K-Akt signaling pathway by several extracellular stimuli, such as growth factors (insulin, BDNF, etc.), affects proliferation, metabolism, and survival in various cell types. In neurons, GSK3β activity and tau phosphorylation and mTOR activity affecting autophagy are influenced. Microglial transit to DAM phenotype, phagocytosis, and cytokine production, and formation of microglia clusters around Aβ plaques are affected. Inflammatory cytokines secreted by microglia activate astrocytes, which transit to the reactive A1 phenotype unable to support neuronal outgrowth and synaptogenesis. Furthermore, genetic variation in *AKT2* gene alters glucose uptake, probably in astrocytes, potentially affecting brain energy metabolism.

The insulin-PI3K-Akt signaling pathway plays an important role in a variety of physiological functions in the brain, such as metabolism, synapse formation, and cell growth and survival. Results from epidemiological, clinical, and animal model-based studies have already established a strong association between T2D and AD, and alteration in PI3K-Akt signaling is the common denominator in these diseases. PI3K subunit (p85 and p110) levels are decreased in AD brain which might have versatile effects in different cell types (Figure 2). Insulin regulates cell growth, apoptosis, autophagy, and protein synthesis in the brain, but plays a minor role there in the control of glucose uptake. An interesting, recent finding revealed that P50T genetic variation in *AKT2* gene, which leads to insulin resistance and hyperinsulinemia in the periphery, increased the glucose uptake in the brain as assessed by [^18^F]-FDG PET imaging (Latva-Rasku et al., 2018). This suggests that specific genetic alterations may exert differential, and also perhaps cell type-specific, functional outcomes in terms of glucose uptake and metabolism. Consistent with this idea, it was recently demonstrated that the signal in [^18^F]-FDG PET imaging does not only represent neuronal glucose uptake but is also strongly affected by the activation of astrocytes (Zimmer et al., 2017). These findings emphasize the role of different cell types and diverse molecular mechanisms underlying the glucose uptake in the brain, but further studies are needed to determine how these novel observations linked to brain glucose uptake and metabolism may mechanistically affect cellular processes relevant for AD pathogenesis. Furthermore, recent findings related to neuroinflammation and TREM2-signaling further underscore the seminal role of the PI3K-Akt pathway in microglia in the context of AD-related pathogenesis. The levels of PI3K subunits p85 and p110 have been shown to be decreased in AD brain (Moloney et al., 2010), which is particularly interesting given that the microglial expression of p85 decreases upon the transition from homeostatic to DAM phenotype (Keren-Shaul et al., 2017). Also, the deficiency of TREM2 in microglia has been shown to impair Akt-mTOR signaling and hence affect autophagy and energy metabolism as well as decrease the ability of microglia to form clusters around Aβ-plaques leading to increased formation of dystrophic neurites (Ulland et al., 2017). These results raise the question whether T2D or life-style factors could affect TREM2-PI3K-Akt signaling and thus the function and activity of microglia.

IN has been shown to activate PI3K-Akt signaling in the brain and to have beneficial effects in individuals with cognitive impairment (Mao et al., 2016; Chapman et al., 2017). IN was also shown to differentially alter the expression of homeostatic microglia markers in AD mice as compared to wild-type mice, suggesting that IN affects the function and activity of microglia depending on the disease status (Gabbouj et al., 2019). Collectively, these genetic and functional findings reinforce the idea that PI3K-Akt signaling cascade in glial cells encompasses a central role in different cellular processes affecting AD pathogenesis beyond its conventional functions in glucose uptake and metabolism. Thus, unraveling the mechanisms in the PI3K-Akt signaling pathway related to altered glial cell function in AD may eventually provide much-needed novel therapeutic targets and treatment strategies for neurodegenerative diseases.

## Author Contributions

SG, AH, MM, MH, and TN designed and outlined the structure and contents of the review. All authors contributed to the literature review, discussion, and writing of the manuscript.

## Conflict of Interest Statement

The authors declare that the research was conducted in the absence of any commercial or financial relationships that could be construed as a potential conflict of interest.
